# 微环境中巨噬细胞、肿瘤新生血管及PD-L1的表达及其与非小细胞肺癌患者预后的关系

**DOI:** 10.3779/j.issn.1009-3419.2020.103.15

**Published:** 2020-10-20

**Authors:** 青青 杭, 航洁 应, 国平 程, 世锋 杨, 佳男 金, 亚梅 陈, 奇勋 陈, 友华 蒋, 强 赵, 敏 方, 明 陈, 霄晶 赖

**Affiliations:** 1 310053 杭州，浙江中医药大学第二临床医学院 The Second Clinical Medical College Zhejiang Chinese Medical University, Hangzhou 310053, China; 2 310022 杭州，中国科学院大学附属肿瘤医院（浙江省肿瘤医院），中国科学院肿瘤与基础医学研究所，浙江省放射肿瘤学重点实验室，胸部放疗科 Zhejiang Key Laboratory of Radiation Oncology, Department of Thoracic Radiotherapy, Cancer Hospital of the University of Chinese Academy of Sciences (Zhejiang Cancer Hospital), Institute of Cancer and Basic Medicine (IBMC), Chinese Academy of Sciences, Hangzhou 310022, China; 3 310022 杭州，中国科学院大学附属肿瘤医院（浙江省肿瘤医院），中国科学院肿瘤与基础医学研究所，浙江省放射肿瘤学重点实验室病理科 Department of Pathology, Cancer Hospital of the University of Chinese Academy of Sciences (Zhejiang Cancer Hospital), Institute of Cancer and Basic Medicine (IBMC), Chinese Academy of Sciences, Hangzhou 310022, China; 4 310022 杭州，中国科学院大学附属肿瘤医院（浙江省肿瘤医院），中国科学院肿瘤与基础医学研究所，浙江省放射肿瘤学重点实验室胸外科 Department of Thoracic Surgery, Cancer Hospital of the University of Chinese Academy of Sciences (Zhejiang Cancer Hospital), Institute of Cancer and Basic Medicine (IBMC), Chinese Academy of Sciences, Hangzhou 310022, China

**Keywords:** 肺肿瘤, 肿瘤微环境, 肿瘤相关巨噬细胞, 程序性死亡受体-配体1, 肿瘤新生血管, Lung neoplasms, Tumor microenvironment, Tumor-associated macrophages, Programmed cell death ligand 1, Tumor neo-vessels

## Abstract

**背景与目的:**

肿瘤微环境是肿瘤细胞赖以生存的复杂环境。其中肿瘤相关巨噬细胞（tumor-associated macrophages, TAMs）、肿瘤新生血管及程序性死亡受体1/程序性死亡受体-配体1（programmed cell death protein 1/programmed cell death ligand 1, PD-1/PD-L1）作为关键部分，在肿瘤发生、发展过程中起重要作用，影响患者预后。本研究旨在阐明TAMs、肿瘤新生血管和PD-L1的表达与非小细胞肺癌（non-small cell lung cancer, NSCLC）临床病理特征的相关性，并探讨它们对NSCLC预后的影响。

**方法:**

收集92例NSCLC患者的临床病理资料及手术标本，采用免疫组化法检测癌组织和癌旁组织中TAMs、肿瘤新生血管和PD-L1的表达，采用配备有Olympus-DP72图像采集系统的Olympus-BX51正置显微镜进行拍照并用Image-pro Plus 6.0软件进行半定量分析。

**结果:**

癌组织与癌旁组织中TAMs、肿瘤新生血管和PD-L1的表达差异无统计学意义（*P* > 0.05）。根据肿瘤微环境中各组分的定量表达，可将其分为低、中、高表达组。癌组织中TAMs的低、中和高密度组的中位总生存（overall survival, OS）分别是36个月（95%CI: 25.3-46.7）、26个月（95%CI: 12.2-39.8）和16个月（95%CI: 9.4-22.6），差异具有统计学意义（*P*=0.016）；肿瘤新生血管的低、中和高密度组的中位OS分别为30个月（95%CI: 22.5-37.5）、28个月（95%CI: 18.1-37.9）和25个月（95%CI: 14.6-35.4），差异无统计学意义（*P*=0.626）；PD-L1的低、中和高表达组的中位OS分别为35个月（95%CI: 29.4-40.6），28个月（95%CI: 13.6-42.4）和17个月（95%CI: 10.5-23.5），差异具有统计学意义（*P*=0.002）。联合低、中和高表达组的中位OS分别为36个月（95%CI: 30.6-41.4）、26个月（95%CI: 19.2-32.8）和9个月（95%CI: 4.4-13.6），差异具有统计学意义（*P*=0.001）。*Cox*回归分析结果显示，病理分型、TAMs和PD-L1均为肺癌患者的独立预后因素。

**结论:**

肿瘤微环境关键成分PD-L1及TAMs的表达与NSCLC患者的预后密切相关。

## Abstract (en)

**Background and Objective:**

Tumor microenvironment is a complex and dynamic community, which plays a crucial role in tumor progression via the co-evolution of cancer cells and tumor stroma. Among them, tumor-associated macrophages (TAMs) and tumor neo-vessels are two key components in the tumor microenvironment during cancer invasion. In addition, programmed cell death ligand 1/programmed cell death ligand 1 (PD-1/PD-L1) also plays an important role in tumorigenesis and development, and the clinical strategies to block PD-1/PD-L1 pathway could have great benefits for cancer patients. This study was aimed at analyzing the quantitative expression and prognostic significance of TAMs, tumor neo-vessels and PD-L1 in tumor microenvironment and exploring the relations between the expression of above components with the patients' prognosis of non-small cell lung cancer (NSCLC).

**Methods:**

Clinico-pathological data and surgical specimens of 92 patients with NSCLC were collected, and immunohistochemistry was used to stain the expression of TAMs, tumor neo-vessels and PD-L1 on tumor tissue and peri-tumor tissues. The inverted microscopy was used to take pictures and Image-pro Plus 6.0 software was used for quantitative analysis. The clinicopathological characteristics and overall survival (OS) were analyzed.

**Results:**

The median OS of 92 NSCLC cases was 22.5 month. The expression of TAMs, tumor neo-vessels and PD-L1 in tumor tissue and peri-tumor tissues were not statistically significant (*P* > 0.05). According to the cutoff of above key three components in tumor microenvironment, all the cases could be classified into high, middle and low expression groups. The survival analysis demonstrated that the OS in high expression group of TAMs (*P*=0.016) and PD-L1 (*P*=0.002) was shorter than the other two groups, respectively, with statistical significance. The OS in high tumor neo vessels group was shorter than the other two groups. However, there was no statistical significance between these three group (*P*=0.626). Combined with above the three components, all the cases could be classified into low, middle and high density groups. The survival analysis demonstrated that the median OS of combined high density group was shorter than the other two groups (*P*=0.001). Multivariate analysis by *Cox* regression indicated that pathological type, TAMs and PD-L1 expression were the independent prognostic factors.

**Conclusion:**

The key components of TAMs and PD-L1 in tumor microenvironment are closely related to the prognosis of NSCLC patients.

肺癌是与癌症相关死亡的最常见原因之一，其中非小细胞肺癌（non-small cell lung cancer, NSCLC）约占85%-90%^[1]^。中国每年约有70万人被诊断为NSCLC，5年总生存率不到15%^[2]^。尽管手术对于可切除的NSCLC是首选治疗方案，但仍有约50%的NSCLC患者在根治性手术后5年内复发或远处转移^[3]^，其根本原因在于肿瘤的侵袭和转移。

肿瘤的侵袭和转移与肿瘤细胞所处的内外环境有着密切关系^[4]^，肿瘤微环境是一个复杂的系统，由细胞外基质（extracellular matrix, ECM）、肿瘤细胞、炎性细胞、免疫浸润细胞、血管内皮细胞、脂肪细胞和成纤维细胞等组成^[5]^。其中，免疫浸润细胞和血管内皮细胞是与肿瘤转移密切相关的两种细胞。肿瘤相关巨噬细胞（tumor-associated macrophages, TAMs）作为免疫细胞浸润的代表，可以与肿瘤细胞相互作用以促进肿瘤的侵袭和转移^[6]^。血管内皮细胞的不成熟和结构异常是肿瘤缺氧的主要原因，它们的不连续性也是肿瘤细胞进入血管发生转移的形态学基础^[7]^。然而，目前关于TAMs和血管内皮细胞对于NSCLC预后的意义却仍不明确，相关研究显示的结果也不一致^[8-10]^。另外，肿瘤新生血管及程序性死亡受体1/程序性死亡受体-配体1（programmed cell death protein 1/programmed cell death ligand 1, PD-1/PD-L1）途径的免疫检查点抑制剂在包括NSCLC在内的多种癌症中已显示出突出的临床疗效^[11]^，但尚未证明其作为独立生物标志物的足够可靠性^[12, 13]^。

因此，本研究通过收集NSCLC患者临床病理资料及手术标本，检测TAMs、肿瘤新生血管及PD-L1在癌组织及癌旁组织中的分布与表达，分析它们与临床病理特征的相关性，并探讨它们对NSCLC预后的影响。

## 1 资料与方法

### 1.1 组织标本及临床资料收取

收集浙江省肿瘤医院自2008年4月-2014年1月NSCLC手术标本92例，包括肿瘤及癌旁组织，癌旁组织为靠近肿瘤2 cm的组织。所有患者均接受了根治性手术治疗，经组织学诊断为NSCLC，且术前未接受任何抗肿瘤治疗。本研究得到浙江省肿瘤医院伦理委员会的批准。通过检索病历构建肺癌临床数据库，包括性别、年龄、吸烟史、病理类型、分化程度和病理性肿瘤原发灶-淋巴结-转移（pathological tumor-node-metastasis, pTNM）分期等临床病理信息。

### 1.2 免疫组化染色（immunohistochemical staining, IHC）

将92例患者的NSCLC组织构建组织芯片，将组织芯片置于二甲苯中脱蜡，用分级酒精水化并进行抗原修复，室温下置于3%过氧化氢（HO）中10 min以灭活内源性过氧化物酶。用PBS洗涤3次后，在室温下用2%牛血清白蛋白（BSA，目录号B2064，西格玛，美国）封闭30 min后，分别滴加一抗CD105（1:500，目录号ab28364，Abcam，英国）、CD68（1:200，目录号ab34710，Abcam，英国）和PD-L1（1:200，目录号：329702，BioLegend，美国）在4 ℃下孵育过夜。用PBS洗涤后，二抗（1:200，目录号ab6721，Abcam，英国或1:200，目录号ab205719，Abcam，英国）在37 ℃下孵育60 min。然后用Dako REALTM EnVision^TM^（DAB，目录号PW017，Sangon生物技术公司，中国）显色5 min，苏木精复染3 min，盐酸酒精分化，反蓝。自来水冲洗1 min后经梯度酒精脱水、透明、最后中性树胶封片。

### 1.3 免疫组织化学染色定量

在配有Olympus DP72相机（Olympus Optical Co.，Ltd.，日本）和CRi Nuance多光谱成像系统（Cambridge Research & Instrumentation，Inc.，美国）的Olympus BX51显微镜下进行拍照，CD68阳性染色为细胞胞质有棕黄（褐）色颗粒；CD105阳性染色为细胞胞浆或胞膜有棕黄（褐）色颗粒；PD-L1阳性染色以细胞胞质染成棕黄（褐）色颗粒。每张切片随机采集400倍图像5张，在获得信号分解图像后，使用Image-pro Plus 6.0软件（IPP, Version 6.0, Media Cybernetics）^[14-16]^，按照HIS选色方案（H=0-30; I=0-255; S=0-255），定义面积范围，过滤50像素以下非特异性的阳性显色杂点，对以CD68标记^[17, 18]^的TAMs、以CD105标记^[19, 20]^的肿瘤新生血管和以PD-L1标记的细胞进行计数，并记录平均细胞计数。根据上述三种成分的定量表达，将各组三等分为低密度组、中密度组和高密度组，CD68、CD105及PD-L1的分组临界值分别为173和251、105和185、62和204。

### 1.4 随访情况

采用复诊登记及电话查询获取随访信息，自手术当天开始计算总生存期，根据超声检查、计算机断层扫描（computed tomography, CT）扫描、磁共振成像（magnetic resonance imaging, MRI）扫描或再次手术确定有无临床复发和转移。末次随访时间是2017年4月，因NSCLC复发转移而死亡为终点事件，因其他原因死亡或失访视为删失数据。其中与肿瘤进展相关死亡92例，明确是否复发转移状态73例。

### 1.5 统计学分析

使用SPSS 25.0进行统计分析。对于所有分析，*P* < 0.05被认为差异具有统计学意义。定性数据通过*χ*^2^检验或*Fisher*精确检验进行比较。计量资料的比较采用*t*检验、*Mann-Whitney*。*Spearman*及*Pearson*相关分析CD68、CD105和PD-L1表达之间的相关性，生存分析采用*Kaplan-Meier*方法，使用*Cox*回归模型执行单因素和多因素分析。

## 2 结果

### 2.1 主要临床病理特征

92例NSCLC患者的具体临床病理特征见表 1，年龄在39岁-75岁之间，中位年龄为61岁，其中男性71例（77.2%），女性21例（22.8%）；66例存在吸烟史（71.7%），26例无吸烟史（28.3%）；肿瘤最大直径≤5 cm的60例（65.2%），32例 > 5 cm（34.8%）；TNM分期I期14例（15.2%），II期28例（30.4%），III期50例（54.3%）；鳞癌52例（56.5%），腺癌40例（43.5%）；低分化16例（55.1%），中分化64例（44.9%）（表 1）。明确复发转移状态的73例患者中，随访到51例复发转移后治疗情况，其中有30例（58.8%）采取两种及以上的综合治疗方法，19例（37.3%）采取单一的治疗方法，2例（3.9%）未采取任何治疗方法。

**1:** 92例NSCLC患者的临床病理特征 Clinicopathological features of 92 NSCLC patients

Category	*n* (%)
Age (yr)	
≤60	41 (44.6)
> 60	51 (55.4)
Gender	
Female	21 (22.8)
Male	71 (77.2)
Tumor size (cm)	
≤5	60 (65.2)
> 5	32 (34.8)
T classification	
T1-T2	62 (67.4)
T3-T4	30 (32.6)
N classification	
N0	30 (32.6)
N1-N2	62 (67.4)
TNM stage	
I-II	42 (45.7)
III	50 (54.3)
Pathological type	
SCC^a^	52 (56.5)
Adenocarcinoma	36 (39.1)
NSCLC other^b^	4 (4.3)
Differentiation degree^*^	
Low	16 (19.8)
Middle	34 (42.0)
Otherc	31 (38.3)
Tumor recurrence^#^	
No	58 (79.5)
Yes	15 (20.5)
Distant metastasis^#^	
No	9 (12.3)
Yes	64 (87.7)
^*^total cases 81. ^#^total cases 73. ^a^SCC: squamous cell carcinoma; ^b^NSCLC other: adenocarcinoma mixed with bronchiolar carcinoma; ^c^other: mixed differentiation degree; NSCLC: non-small cell lung cancer; TNM: tumor-node-metastasis.	

### 2.2 肺癌微环境中CD68、CD105和PD-L1的分布和定量表达分析

CD68、CD105和PD-L1染色主要位于细胞质或细胞膜上，在大多数肿瘤细胞中，PD-L1表达较低，少数高表达。92例NSCLC肿瘤组织和癌旁组织中CD68阳性细胞数平均值分别为（208±86）个和（190±84）个；CD105阳性细胞数平均值分别为（156±84）个和（143±69）个。肿瘤组织和癌旁组织中PD-L1阳性细胞平均值分别为（151±125）个和（136±100）个。CD68、CD105和PD-L1在癌组织及癌旁组织中的表达无统计学差异（*P* > 0.05），但其在肿瘤微环境中的表达和分布均存在异质性（图 1）。研究结果显示肿瘤组织中PD-L1的表达与CD68的表达显著相关（*Pearon*相关系数*r*=0.227，*P*=0.030），而PD-L1与CD105之间的相关性无显著统计学意义（*Pearson*相关系数*r*=0.002，*P*=0.986），同时CD68与CD105的表达存在显著相关性（*Pearson*相关系数*r*=0.240，*P*=0.021）。

**1:**
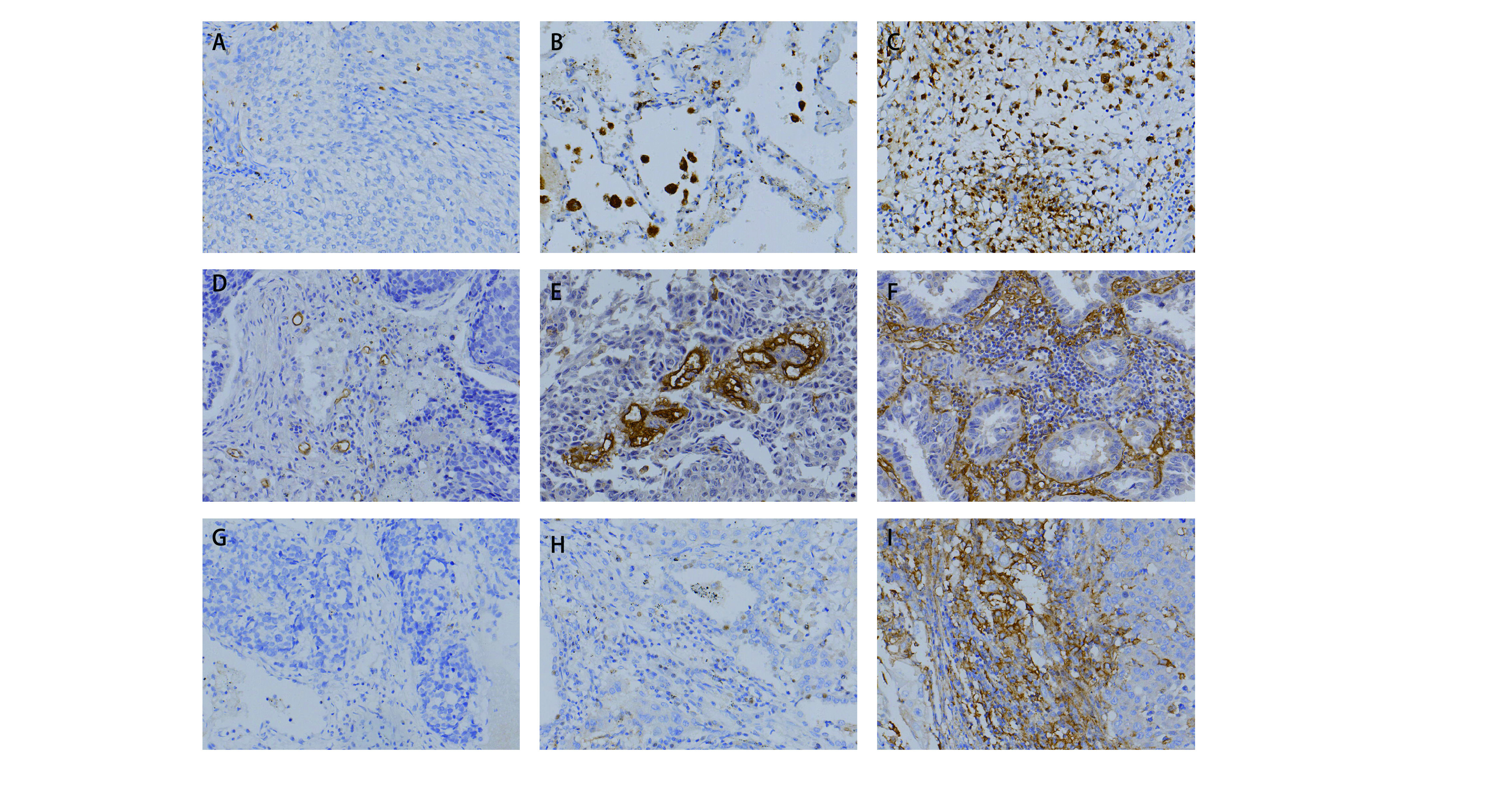
NSCLC组织中TAMs、肿瘤新生血管和PD-L1的表达情况。A-C：分别为以CD68标记的TAMs在肿瘤组织中的低、中、高表达；D-F：分别为以CD105标记的肿瘤新生血管的低、中、高表达；G-I：分别为PD-L1的低、中、高表达（400×）。 The expression of TAMs, tumor neo-vessels and PD-L1 in NSCLC tumor tissues. A-C: the low, middle and high expression of TAMs labeled with CD68. D-F: low, middle and high expression of tumor neo-vessels labeled with CD105; G-I: low, middle and high expression of PD-L1 respectively (400×).

### 2.3 生存分析

在92例NSCLC病例中，中位OS为22.5个月，且CD68和PD-L1的表达与OS呈负相关（*Spearman*相关系数*r*=-0.340，*P*=0.001和*Spearman*相关系数*r*=-0.268，*P*=0.010）。将肿瘤微环境中上述三种成分进行联合分析，三个成分均为低密度组分为联合低表达组；三个成分均为高密度组分为联合高表达组；其余为联合中表达组。生存分析曲线显示（图 2），CD68表达的低、中和高密度组的中位OS分别是36个月（95%CI: 25.3-46.7）、26个月（95%CI: 12.2-39.8）和16个月（95%CI: 9.4-22.6），差异具有统计学意义（*P*=0.016）；CD105表达的低、中和高密度组的中位OS分别为30个月（95%CI: 22.5-37.5）、28个月（95%CI: 18.1-37.9）和25个月（95%CI: 14.6-35.4），差异无统计学意义（*P*=0.626）；PD-L1表达的低、中和高表达组的中位OS分别为35个月（95%CI: 29.4-40.6）、28个月（95%CI: 13.6-42.4）和17个月（95%CI: 10.5-23.5），差异具有统计学意义（*P*=0.002）。联合低、中和高表达组的中位OS分别为36个月（95%CI: 30.6-41.4）、26个月（95%CI: 19.2-32.8）和9个月（95%CI: 4.4-13.6），差异具有统计学意义（*P*=0.001）（表 2）。

**2:**
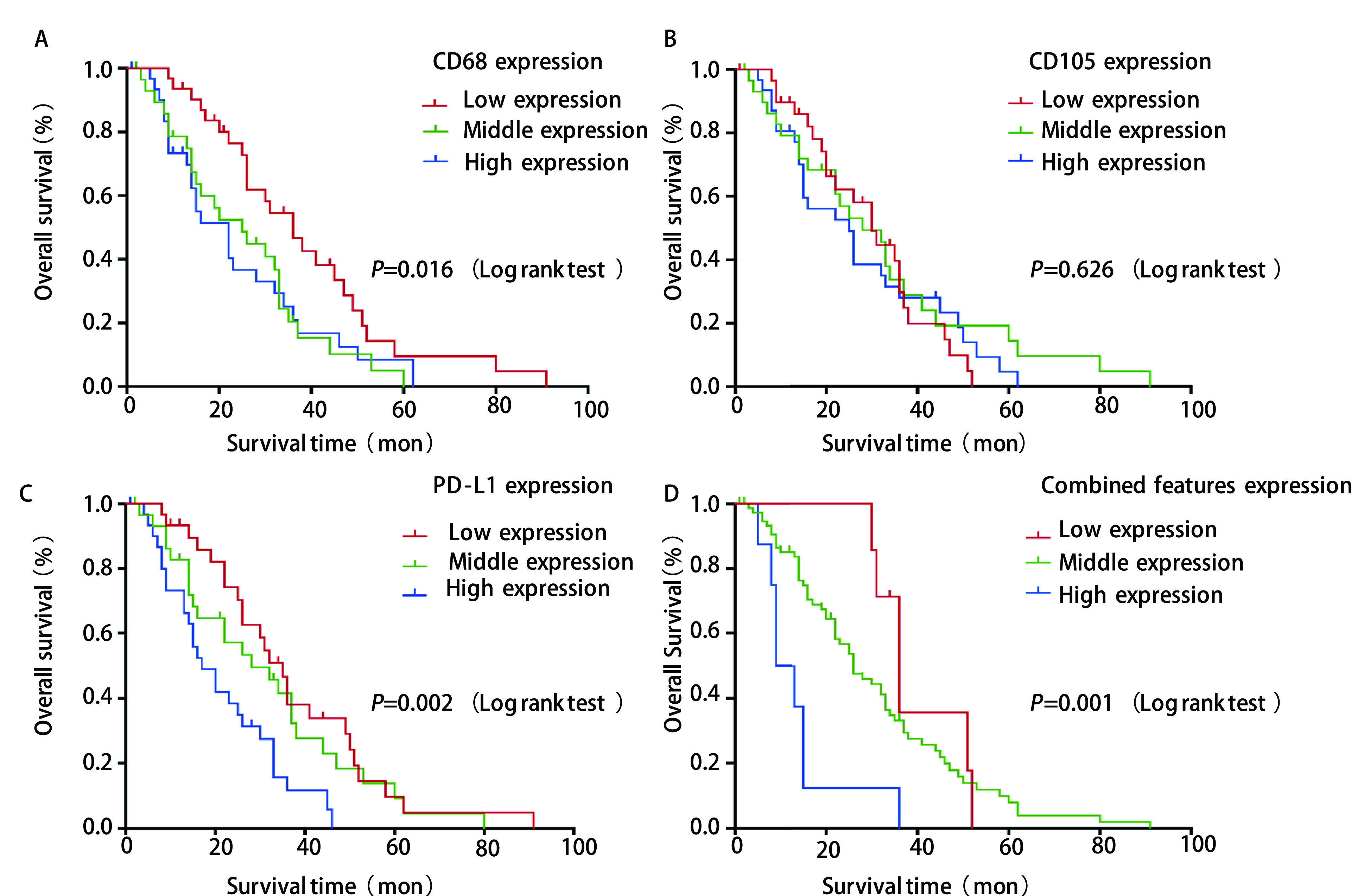
生存分析。A-D分别是CD68、CD105、PD-L1及联合分析的低中高表达组的92例术后非小细胞肺癌患者生存分析。 *Kaplan-Meier* curves. A-D: Kaplan-Meier curves for 92 NSCLC patiens according to CD68, CD105, PD-L1 and combined features, respectively.

**2:** CD68、CD105及PD-L1的表达与预后的关系 Relationship between the expression of CD68, CD105 and PD-L1 with prognosis

Item	*n* (%)	Median overall survival (mon)	*χ*^2^	*P*
CD68 expression			8.240	0.016
Low	31 (33.7)	36.0		
Middle	30 (32.6)	26.0		
High	31 (33.7)	16.0		
CD105 expression			0.937	0.626
Low	31 (33.7)	30.0		
Middle	30 (32.6)	28.0		
High	31 (33.7)	25.0		
PD-L1 expression			12.184	0.002
Low	31 (33.7)	35.0		
Middle	30 (32.6)	28.0		
High	31 (33.7)	17.0		
Combined features			13.926	0.001
Low	7 (7.6)	36.0		
Middle	77 (83.7)	26.0		
High	8 (8.0)	9.0		

### 2.4 多因素分析

在单因素分析中，分化程度、T分期、CD68和PD-L1的表达与OS有关。此外，对CD68、CD105和PD-L1等肿瘤微环境关键成分进行联合分析表明，联合高表达组的死亡风险显著增加（*P*=0.001）。如表 3所示，将单因素分析中*P* < 0.2因子纳入多因素分析，结果显示CD68和PD-L1的表达是OS的独立预后因素（*P* < 0.05）。

**3:** NSCLC预后单因素和多因素*Cox*风险比例模型分析（*n*=92） Univariate and multivariate *Cox* proportional hazards model analyses of OS for NSCLC (*n*=92)

Index	Comparison	Univariate analysis			Multivariate analysis	
		HR (95%CI)	*P*		HR (95%CI)	*P*
Gender	Female *vs* Male	0.980 (0.572-1.678)	0.941			
Age	> 60 yr *vs* ≤60 yr	1.269 (0.804-2.005)	0.306			
Smoking status	No *vs* Yes	1.162 (0.698-1.932)	0.564			
Tumor size	≥5 cm *vs* < 5 cm	1.373 (0.850-2.218)	0.195		1.338 (0.755-2.370)	0.319
Differentiation degree	Middle *vs* Low	0.827 (0.431-1.590)	0.570			
	Other *vs* Low	0.472 (0.242-0.920)	0.028		0.414 (0.200-0.857)	0.018
T classification	T1-T2 *vs* T3-T4	1.614 (1.001-2.603)	0.049		0.865 (0.454-1.647)	0.658
N classification	N0 *vs* N1-N2	0.903 (0.562-1.452)	0.674			
TNM stage	Early *vs* Advanced^b^	1.123 (0.706-1.785)	0.624			
Pathological type	Adenocarcinoma *vs* SCC	0.714 (0.444-1.149)	0.165			
	NSCLC other *vs* SCC	0.323 (0.099-1.057)	0.062		0.164 (0.040-0.664)	0.011
CD68 expression	Continuous numeric variable	1.004 (1.001-1.007)	0.005		1.006 (1.002-1.010)	0.006
CD105 expression	Continuous numeric variable	1.001 (0.998-1.004)	0.460		1.001 (0.997-1.005)	0.608
PD-L1 expression	Continuous numeric variable	1.003 (1.002-1.005)	< 0.001		1.002 (1.000-1.004)	0.047
^b^ Early: TNM stage I/II; advanced: TNM stage III; OS: overall survival; HR: hazard ratio; CI: confidence interval.						

## 3 讨论

人们普遍认为，包括NSCLC在内的肿瘤侵袭不仅是一个局部问题，而且是一个多因素、多阶段逐步发展的过程，存在多种分子功能障碍和细胞信号转导失调。肿瘤细胞的遗传或表观遗传改变是肿瘤发生的“初始因素”，肿瘤微环境中间质细胞的反应可“促进”或“调节”肿瘤的侵袭和转移，最终导致肿瘤微环境向有利于肿瘤侵袭和转移的方向发展，影响患者预后。肿瘤细胞与其微环境之间的相互作用在肿瘤发展的各个方面都至关重要，包括肿瘤细胞的休眠、增殖、侵袭和迁移过程。肿瘤炎症和肿瘤血管生成被认为是癌症侵袭过程中必不可少的。TAMs作为肿瘤微环境中主要的免疫细胞能够诱导机体炎症反应，协助肿瘤细胞逃脱免疫监视，增强肿瘤细胞的迁移和侵袭能力^[21]^。异常的、不成熟的新生血管在大多数肿瘤的发展过程中显著增加，它们的形成可以维持快速生长的肿瘤组织的血液流动，为肿瘤细胞提供营养和氧气^[22]^。本研究证实了NSCLC患者不良预后与TAMs有关，但与肿瘤新生血管的形成无显著相关性。同时本研究表明了肿瘤新生血管与TAMs存在一定关联，三种成分联合分析与NSCLC患者预后有关。可以推测肿瘤新生血管在肿瘤侵袭转移过程中，需要借助肿瘤微环境的其他关键成分共同发挥作用。

PD-L1的表达与NSCLC患者预后的关系目前报道不一。有研究^[23]^显示PD-L1高表达可使早期NSCLC患者获得更长的生存期。也有许多研究^[24-26]^发现，PD-L1高表达与NSCLC不良预后有关，与本研究结果一致。而另一项对接受化疗的晚期NSCLC患者的研究^[27]^却显示PD-L1表达与总生存率之间无相关性。相关研究显示PD-L1的表达实际上是一种适应性机制，与内源性免疫反应相关^[28]^，可能是肿瘤细胞对宿主免疫压力的反应^[29]^。

本研究还证实了PD-L1的表达与TAMs相关，这与一项在胃腺癌的研究^[30]^中得到的研究结果一致。TAMs可以促进肿瘤免疫抑制环境，其分泌的免疫抑制性细胞因子或蛋白酶会诱导PD-L1在癌细胞中的过度表达并激活相关的信号通路，加速肿瘤进程^[31]^。有研究^[32]^显示NSCLC中的M2型巨噬细胞，其表面表达PD-L1，并直接抑制T细胞反应。此外，也有研究报道了肿瘤细胞可以诱导巨噬细胞向M2型极化并增加PD-L1的表达^[33]^。

总之，肿瘤微环境中的关键分子与肿瘤细胞相互作用，共同促进着肿瘤的侵袭和转移。任何可能的预后意义均不与单个免疫信号直接相关，而是与宿主抗肿瘤免疫反应和肿瘤介导的免疫抑制之间的总体平衡有关。TAMs与PD-L1或许能成为预测NSCLC术后总生存的预测因子。本研究中TNM分期与患者预后无关，产生差异的原因在于病例非连续入组，存在选择偏倚，近年来对于NSCLC根治术后预防复发转移的治疗方法多样且复发转移后治疗方法不同，影响患者预后。本研究随访资料还不完善，病例数较少后续仍然需要扩大样本量进行进一步研究，从而更深入地认识和解释这种机制。
